# Foleys protection caps: inexpensive alternative in Ilizarov fixation

**DOI:** 10.1308/003588412X13373405387096e

**Published:** 2012-05

**Authors:** K Abbas, H Rashid

**Affiliations:** Aga Khan University Hospital, Karachi,Pakistan

**Figure 1:**
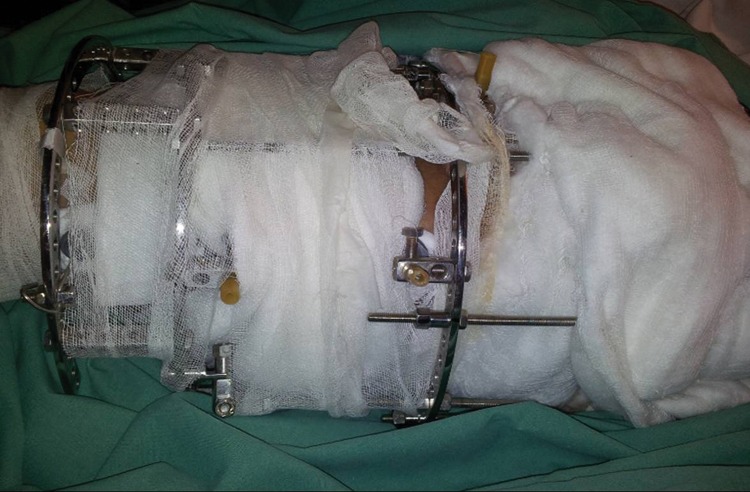
Foley catheter cut and rolled over the sharp ends of Schanz screws on an Ilizarov ring fixator

Use of a hybrid type of Ilizarov ring fixator is popular in complex orthopaedic trauma, typically comprising Schanz screws at each ring and tensioned wires. The Schanz screws are cut to the size of the ring but their cut ends may subsequently injure the other leg or damage garments and bed sheets. Custom made protective caps are available but a good alternative is a 22/24Fr Foley catheter, cut and rolled over the sharp ends of screws (Fig 1). These are readily available in theatres and provide remarkable protection even when direct pressure is applied over the cut screw ends.

